# Attenuation of *Aggregatibacter actinomycetemcomitans* virulence using curcumin-decorated nanophytosomes-mediated photo-sonoantimicrobial chemotherapy

**DOI:** 10.1038/s41598-021-85437-6

**Published:** 2021-03-16

**Authors:** Maryam Pourhajibagher, Abbas Bahador

**Affiliations:** 1 Dental Research Center, Dentistry Research Institute, Tehran University of Medical Sciences, Tehran, Iran; 2 Oral Microbiology Laboratory, Department of Microbiology, School of Medicine, Tehran University of Medical Sciences, Tehran, Iran; 3 Fellowship in Clinical Laboratory Sciences, Iran University of Medical Sciences, Tehran, Iran

**Keywords:** Antimicrobials, Biofilms, Microbiology, Molecular biology

## Abstract

This study aimed to focus on the simultaneous use of antimicrobial photodynamic therapy (aPDT) and sonodynamic antimicrobial chemotherapy (SACT), which is called photo-sonodynamic antimicrobial chemotherapy (PSACT) to attenuate the virulence of *Aggregatibacter actinomycetemcomitans*. Following the synthesis of Curcumin-decorated nanophytosomes (Cur-NPhs) as a novel photo-sonosensitizer, its particle size, polydispersity, ζ-potential surface morphology, physical stability, drug release, and entrapment efficiency were determined. In the Cur-NPhs-PSACT, the antimicrobial activities of Cur-NPhs against *A. actinomycetemcomitans* were investigated using cell viability, biofilm killing/degradation, metabolic activity, expression of quorum-sensing-associated *qseB* and *qseC* genes, and biofilm-associated *rcpA* gene under blue laser irradiation plus ultrasonic waves. Characterization tests showed the presence of a sphere-shaped vesicle and the self-closed structure of Cur-NPhs, resulting in a high drug-loading content and encapsulation efficiency. However, the antimicrobial effect of Cur-NPhs-PSACT was dose-dependent, PSACT using the high concentrations of Cur-NPhs (50 × 10^–4^ g/L) showed significant reductions (P < 0.05) in cell viability (13.6 log_10_ CFU/mL), biofilm killing/degradation (65%), metabolic activity (89.6%,), and mRNA levels of virulence determinant genes (*qseB*; 9.8-fold, *qseC*; 10.2-fold, and *recA*; 10.2-fold). This study concludes that the Cur-NPhs-PSACT had antimicrobial activities against *A. actinomycetemcomitans* by downregulating the expression of virulence genes, and may attenuate this bacterium that decreases periodontal disease severity in patients.

## Introduction

Periodontal disease is a collective form of the inflammatory disorder that is induced by oral microorganisms, dental plaque, or dental biofilm. According to the latest Portuguese Oral National Health Survey (PONHS), the prevalence of periodontitis was 10.8% and 15.3% in adults and the elderly, respectively^1^. Several factors that increase the risk of periodontal diseases are divided into two groups; 1. Modifiable risk factors such as smoking, poor oral hygiene, hormonal changes in females, diabetes mellitus, medications, and stress, 2. Non-modifiable risk factors including age and hereditary^2^. In addition, microbial pathogens are one of the most common causative agents of periodontal disease^3^.

*Aggregatibacter actinomycetemcomitans* is one of the main causes of periodontal disease in juveniles and adolescents^4^. The pathology and etiology of periodontal disease have demonstrated several virulence factors such as lipopolysaccharide (LPS), fimbriae, and enzymes can trigger inflammation in periodontal tissues caused by *A. actinomycetemcomitans*^5^.

The success of periodontitis treatment depends on eliminating the bacterial load of periodontal pockets to restore biological compatibility of periodontally diseased root surfaces^6^. The treatment of periodontitis is challenging and routine mechanical or non-surgical periodontal treatments do not diminish effectively the load of the microbial strains. On the other hand, the over-use of systemic and local antibiotics has been the main reason for the emergence of drug-resistant bacteria^7^. Nevertheless, the application of the alternative approaches for the removal of periopathogens from periodontal pockets is highly essential. Therefore, the development of a new antimicrobial approach with fewer complications is necessary.

Antimicrobial photodynamic therapy (aPDT) and sonodynamic antimicrobial chemotherapy (SACT) as the non-invasive therapeutic modalities are the new antimicrobial strategies to improve periodontal treatment^8^. SACT is analogous to aPDT, except that drug activation is achieved through ultrasound instead of visible light. As well, photosensitizer as a photosensitizing agent in aPDT is replaced by sonosensitizer as a sonosensitizing agent in SACT^9^. The antimicrobial effects of aPDT and SACT depend on the production of cytotoxic reactive oxygen species (ROS) including singlet oxygen (^1^O_2_), hydroxyl radicals (·OH), peroxyl radical (ROO·), and superoxide (O_2_^−^) ions causing damage to the cellular and molecular structures of microorganisms^10^.

In the present study, for the first time, we aim to examine the antimicrobial effects of the simultaneous use of aPDT and SACT, which called photo-sonodynamic antimicrobial chemotherapy (PSACT) using Curcumin-decorated nanophytosomes (Cur-NPhs) as the photo-sonosensitizer on the regulation of *qseB* and *qseC* genes expression in *A. actinomycetemcomitans*. After confirmation of Cur-NPhs synthesis, we examined the effects of Cur-NPhs-mediated PSACT on cell viability, biofilm degradation, metabolic activity, and *qseB* and *qseC* genes expression.

By revealing the interaction of suppression of metabolic activities during treatments with suppression of *rcpA* expression among *A. actinomycetemcomitans*, we attempt to explain, at least in part, the suppression of *rcpA* expression is likely a byproduct of the general suppression of metabolic activities, and the Cur-NPhs-mediated PSACT targeted specifically the bacterial biofilm structure. Our findings might provide additional options for the management of periodontitis and/or pri-implantitis, either as direct treatment strategies or adjuncts to routine methods.

## Results

### Confirmation of synthesized Cur-NPhs

**Figure 1:**
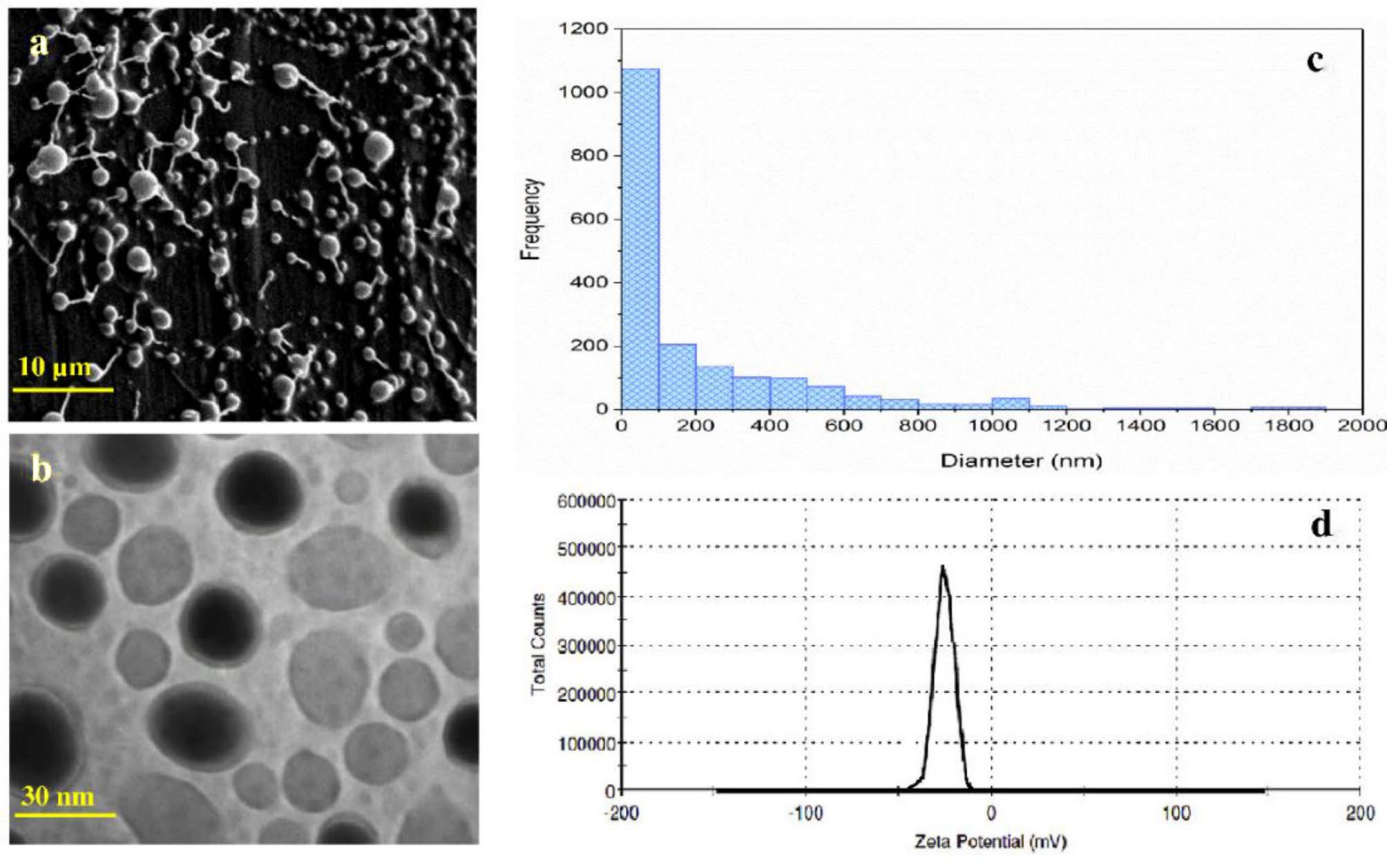
Characterization of synthesized Cur-NPhs. (**a**) Representative scanning electron micrograph (scale bar represents 10 μm). (**b**) Representative transmission electron micrograph (scale bar represents 30 nm). (**c**) Average particle size. (**d**) ζ-potential value.

As shown in Fig. 1a, the size of the Cur-NPhs was 156.42 ± 2.06 nm and the polydispersity index was 0.34 ± 0.02. The presence of a sphere-shaped vesicle and the self-closed structure was indicated in the TEM of the Cur-NPhs complex (Fig. 1b). It was observed that the average particle size and the ζ-potential value were 86.3 ± 2.08 nm and − 34.7 ± 1.3 mV, respectively, which indicate good stability (Fig. 1c,d).

High EE is the main factor for the choice of a specific carrier and the encapsulation approach. According to the results, the EE percentage of the Cur in Cur-NPhs was 83.56 ± 0.5%.

### In vitro drug release

**Figure 2:**
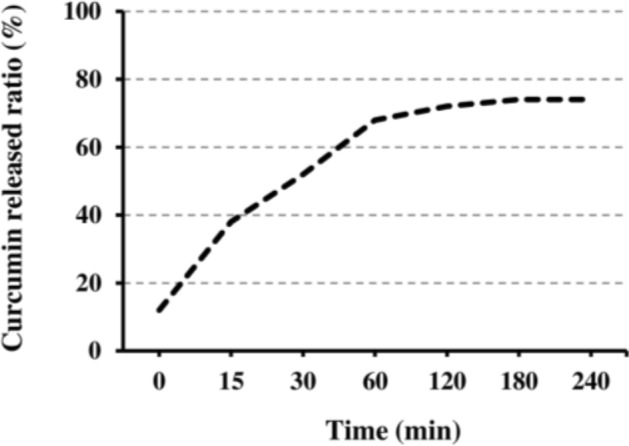
Profile of Cur release from Cur-NPhs.

The release profile showed the release of Cur occurred in a biphasic process in which a rapid initial burst of about 12% within the first hour followed by sustained drug release of about 74% in 4 h (Fig. 2). The drug entrapped on the surface of the phytosomes might be the reason for the observed initial burst release.

### Physical stability of Cur-NPhs

**Figure 3:**
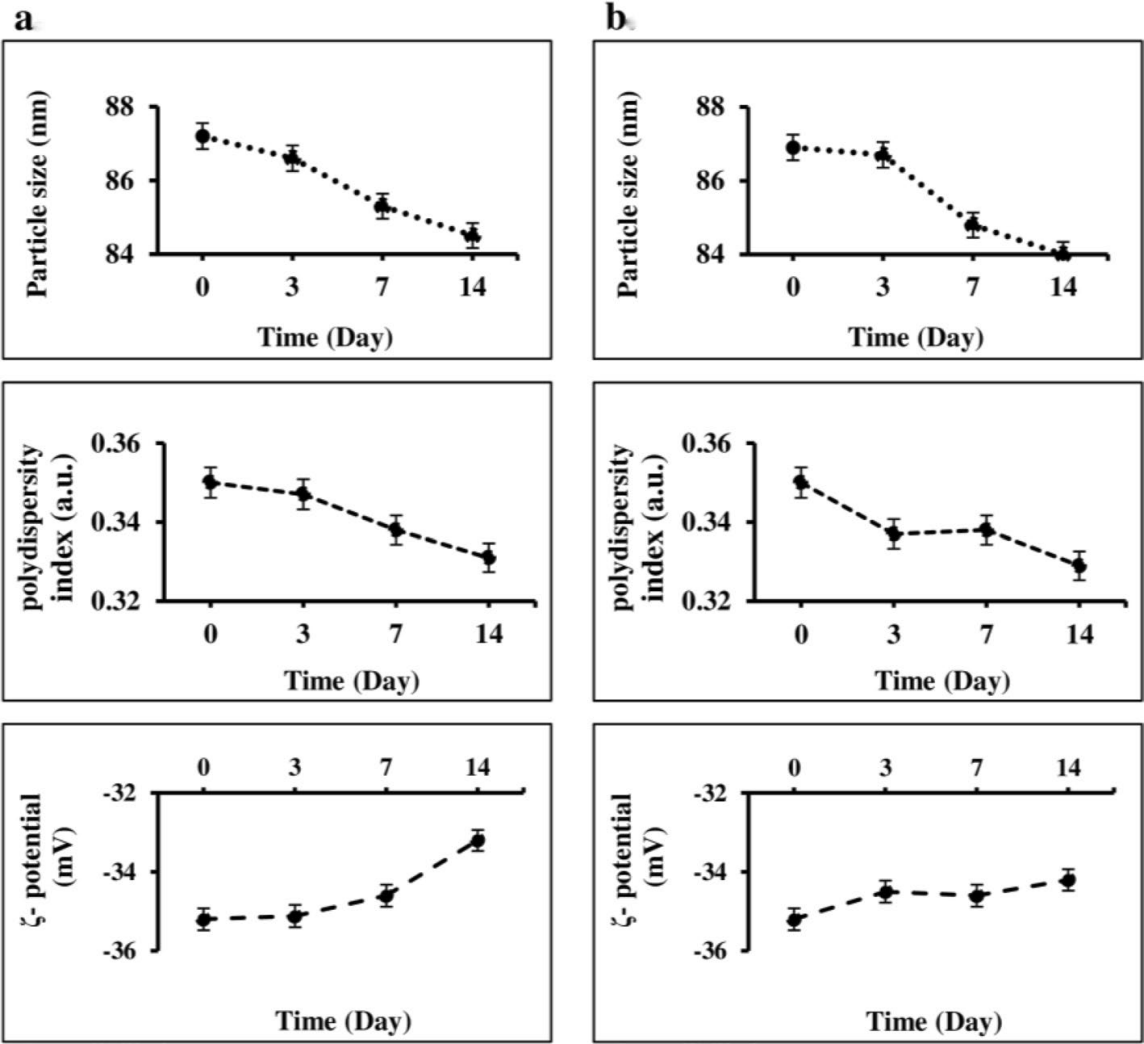
Particle size, polydispersity index, and ζ-potential of Cur-NPhs in different conditions; (**a**) at 4 °C, (**b**) at 37 °C.

Cur-NPhs stability was measured by particle size over 2 weeks at 4 °C and 37 °C, which showed no significant variation between day 0 and day 14 (P > 0.05; Fig. 3). The results showed no significant changes in polydispersity index and ζ-potential of Cur-NPs during 2 weeks.

**Figure 4:**
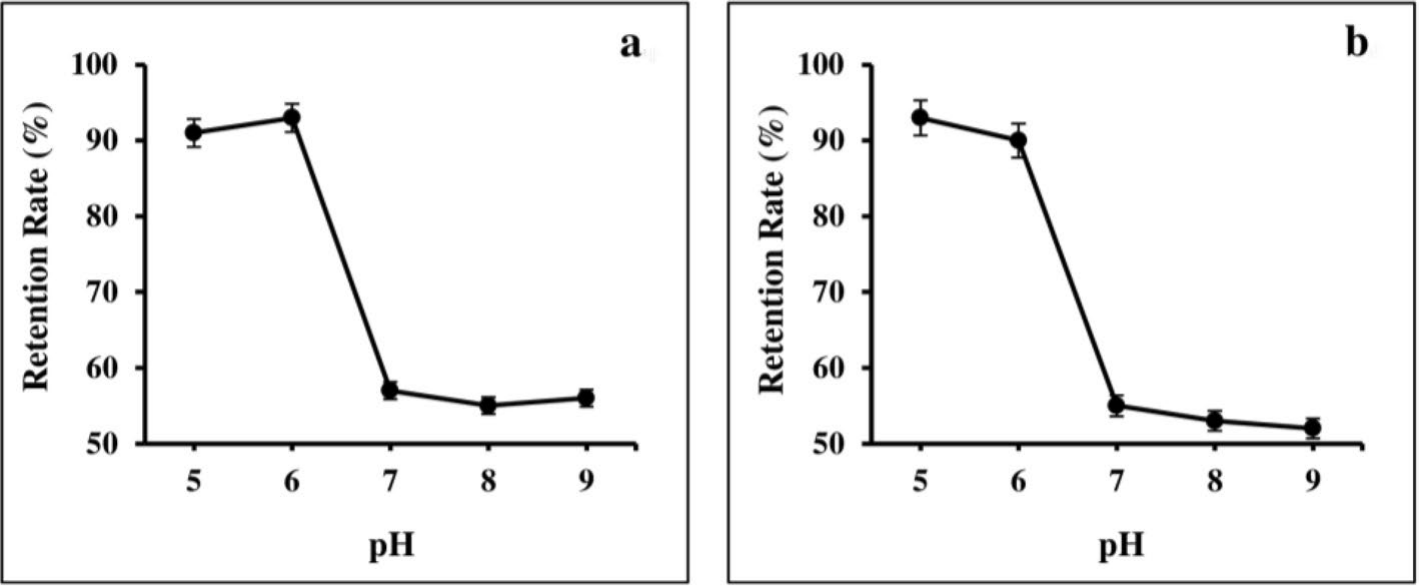
Retention rate percentage of Cur-NPhs at different pH; (**a**) during 24 h and (**b**) 48 h storage period.

Also, the effects of different pH treatments on the RR of Cur-NPs were shown in Fig. 4. During the 24 and 48 h storage period, Cur-NPhs were more stable under acidic conditions with RR more than 90%, whiles RR of Cur-NPhs decreased to 55% at neutral and alkaline conditions. Meanwhile, no significant changes were achieved in RR of Cur-NPhs after 48 h of storage (P > 0.05). It is therefore concluded that Cur-NPs did not undergo degradation on storage.

### Cytotoxicity effects of treatment groups on *A. actinomycetemcomitans* viability

**Figure 5:**
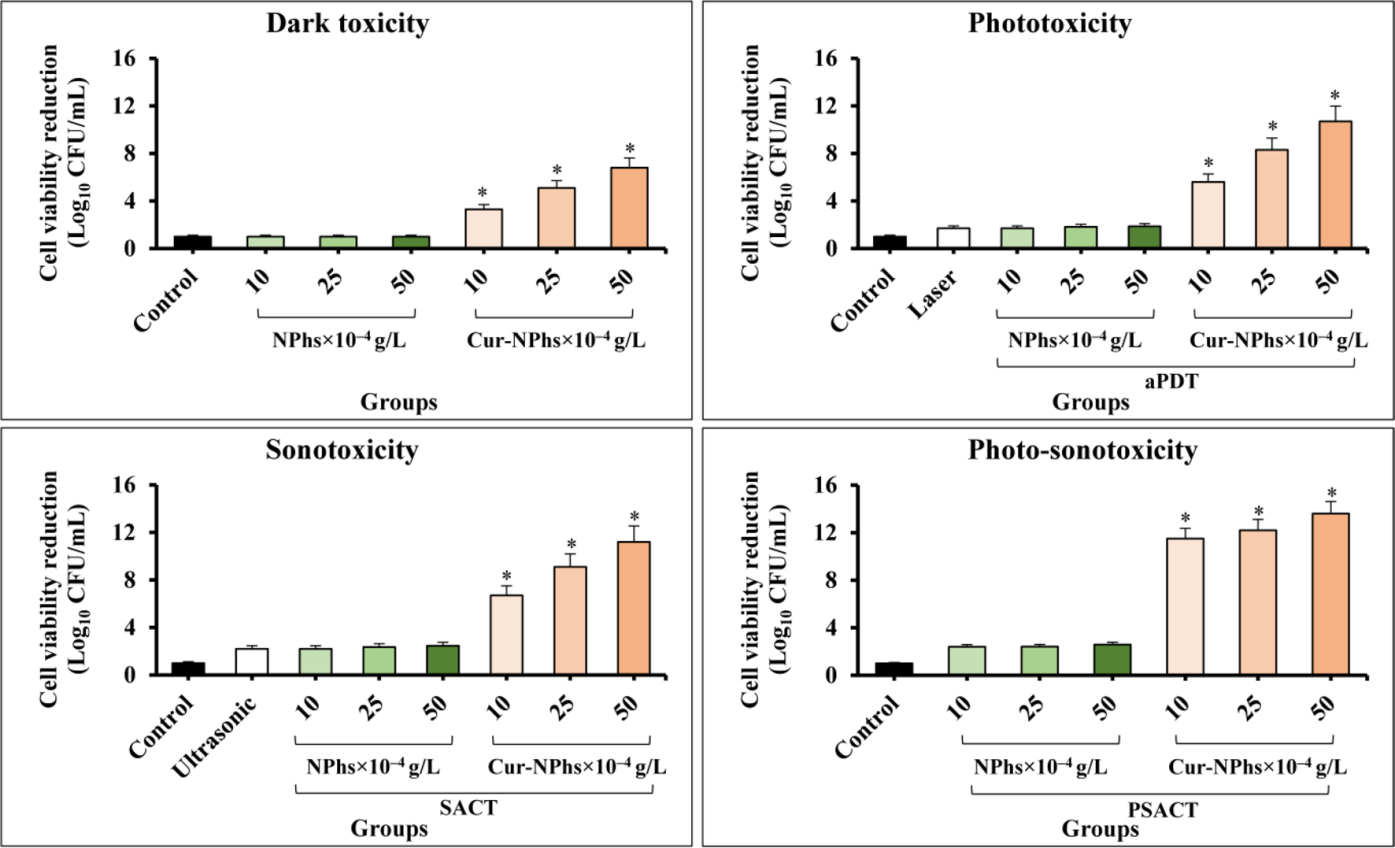
Cell viability of *A. actinomycetemcomitans* following different treatments. Significant differences according to the control, *P < 0.05.

A dose-dependent decrease was observed in log_10_ CFU/mL of *A. actinomycetemcomitans* (Fig. 5). The log_10_ CFU/mL reduction was increased to 3.3 ± 0.06, 5.1 ± 0.08, and 6.8 ± 0.05, after treatment with 10, 25, and 50 × 10^–4^ g/L Cur-NPhs, respectively (P < 0.05). As shown in Fig. 5, cell viability significantly decreased in treated *A. actinomycetemcomitans* with aPDT and SACT using different concentrations of Cur-NPhs compared with the control group (P < 0.05). The results indicated that 50 × 10^–4^ g/L Cur-NPhs plus 2 min blue laser irradiation in aPDT group and 50 × 10^–4^ g/L Cur-NPhs plus ultrasonic waves with the intensity of 1.56 W/cm^2^ in SACT group could effectively decrease 10.7 ± 0.05 log_10_ CFU/mL and 11.2 ± 0.03 log_10_ CFU/mL of microbial cells, respectively (P < 0.05). Interestingly, PSACT was able to reduce the survival rate of bacteria more than the other groups compared to the control group (P < 0.05), so that a 13.6 ± 0.08 log_10_ CFU/mL reduction was observed in 50 × 10^–4^ g/L Cur-NPhs-PSACT-treated cells (Fig. 5).

According to the data obtained in the present study, there was no significant difference in the rate of *A. actinomycetemcomitans* viability following NPhs, NPhs-mediated aPDT-, SACT-, and PSACT-treatment, blue laser irradiation at a wavelength of 450 ± 5 nm for 2 min, as well as, ultrasonic waves with the intensity of 2 W/cm^2^ for 2 min at a frequency of 1 MHz (P > 0.05).

### Anti-biofilm potency of treatment groups against *A. actinomycetemcomitans*

**Figure 6:**
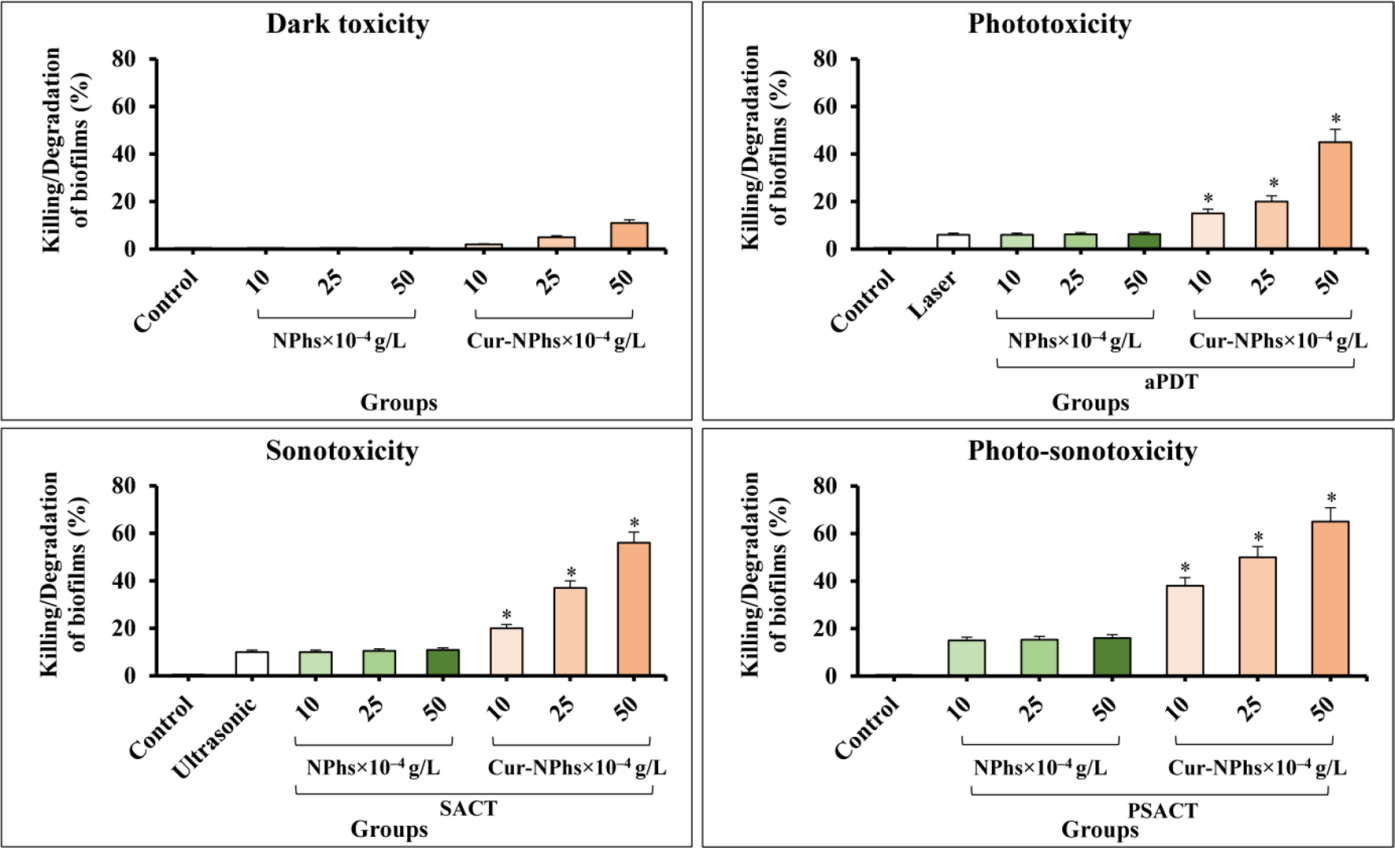
Biofilm degradation ability of *A. actinomycetemcomitans* following different treatments. Significant differences according to the control, *P < 0.05.

The results of the CV assay showed that aPDT, SACT, and PSACT with different concentrations of Cur-NPhs decreased the biofilm forms of *A. actinomycetemcomitans* compared to that of untreated biofilm (P < 0.05; Fig. 6). According to the results, degradation of *A. actinomycetemcomitans* biofilms was dose-dependent. PSACT using 50 × 10^–4^ g/L Cur-NPhs plus ultrasound waves with the intensity of 1.56 W/cm^2^ was the most effective biofilm degradation against *A. actinomycetemcomitans* (65% biofilm killing; P < 0.05). In contrast, there was no considerable degradation in biofilms of *A. actinomycetemcomitans* exposed to the NPhs, Cur-NPhs, blue laser irradiation, ultrasonic waves alone, NPhs-mediated aPDT-, SACT-, and PSACT-treatment (P > 0.05).

### Determination of *A. actinomycetemcomitans* metabolic activity following treatment groups

**Figure 7:**
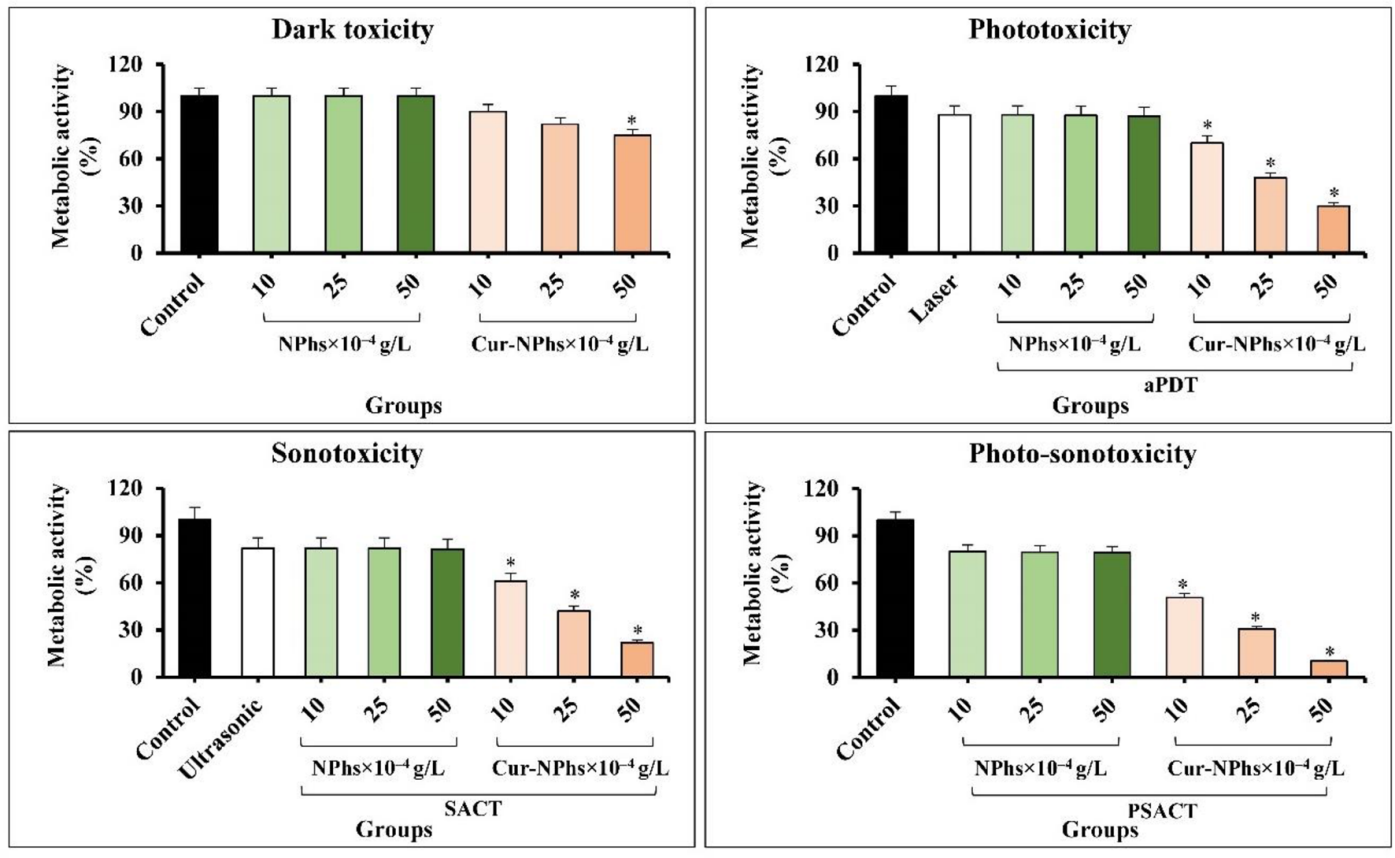
Metabolic activity of *A. actinomycetemcomitans* following different treatments. Significant differences according to the control, *P < 0.05.

Although considerable reduction of *A. actinomycetemcomitans* metabolic activity was observed upon treatment with Cur-NPs-aPDT and Cur-NPs-SACT, therapy employing Cur-NPs-PSACT generated a significant reduction of metabolic activity more than other groups (Fig. 7; P < 0.05). There was a more significant reduction of metabolic activity following Cur-NPhs-PSACT at the concentrations of 10, 25, and 50 × 10^–4^ g/L to 49.3%, 69.2%, and 89.6%, respectively (all P < 0.05; Fig. 7). In contrast, no significant changes were observed in metabolic activity of *A. actinomycetemcomitans* after exposure with blue laser irradiation, ultrasonic waves, and different concentrations of NPs and Cur-NPhs alone (P > 0.05), except 50 × 10^–4^ g/L Cur-NPhs (P < 0.05). Also, there was no significant effect in metabolic activity of *A. actinomycetemcomitans* following NPhs-mediated aPDT-, SACT-, and PSACT-treatment (P > 0.0.5).

### Monitoring of quorum-sensing (QS)-associated *qseB* and *qseC* genes expression

**Figure 8:**
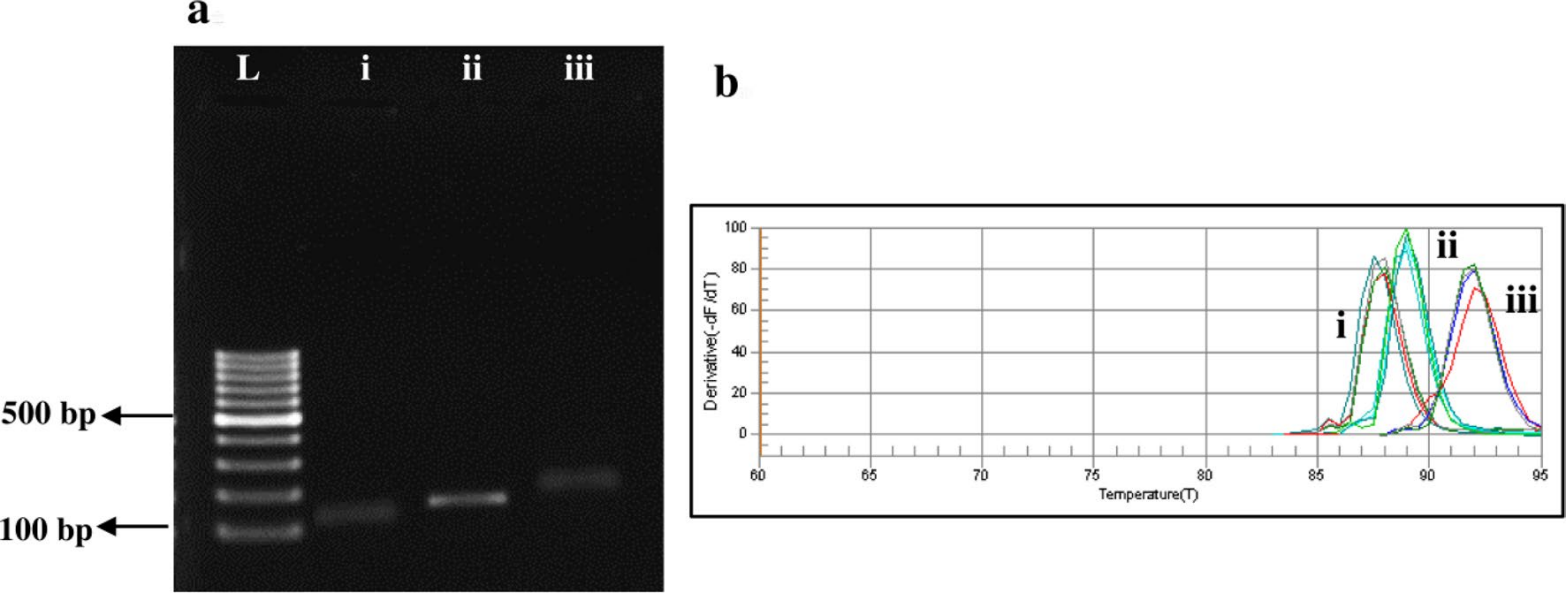
Specificity of the designed primers; a. A 2% agarose gel electrophoresis for detection of *qseB, qseC,* and *16S rRNA*. *L* ladder 100 bp, i: *qseB*, ii: *qseC*, iii: *16S rRNA*. b. Melting curve, i: *qseB*, ii: *qseC*, iii: *16S rRNA*.

The agarose gels of the amplified product showed the single bands corresponding to the predicted amplicon length. The amplification of *qseB*, *qseC*, and *16S rRNA* yielded no false-positive and unspecific bands in test samples (Fig. 8). As shown in Fig. 8, dissociation melting curves of *qseB*, *qseC*, and *16S rRNA* amplicons with a single peak demonstrates the specificity of the designed primer pairs.

**Figure 9:**
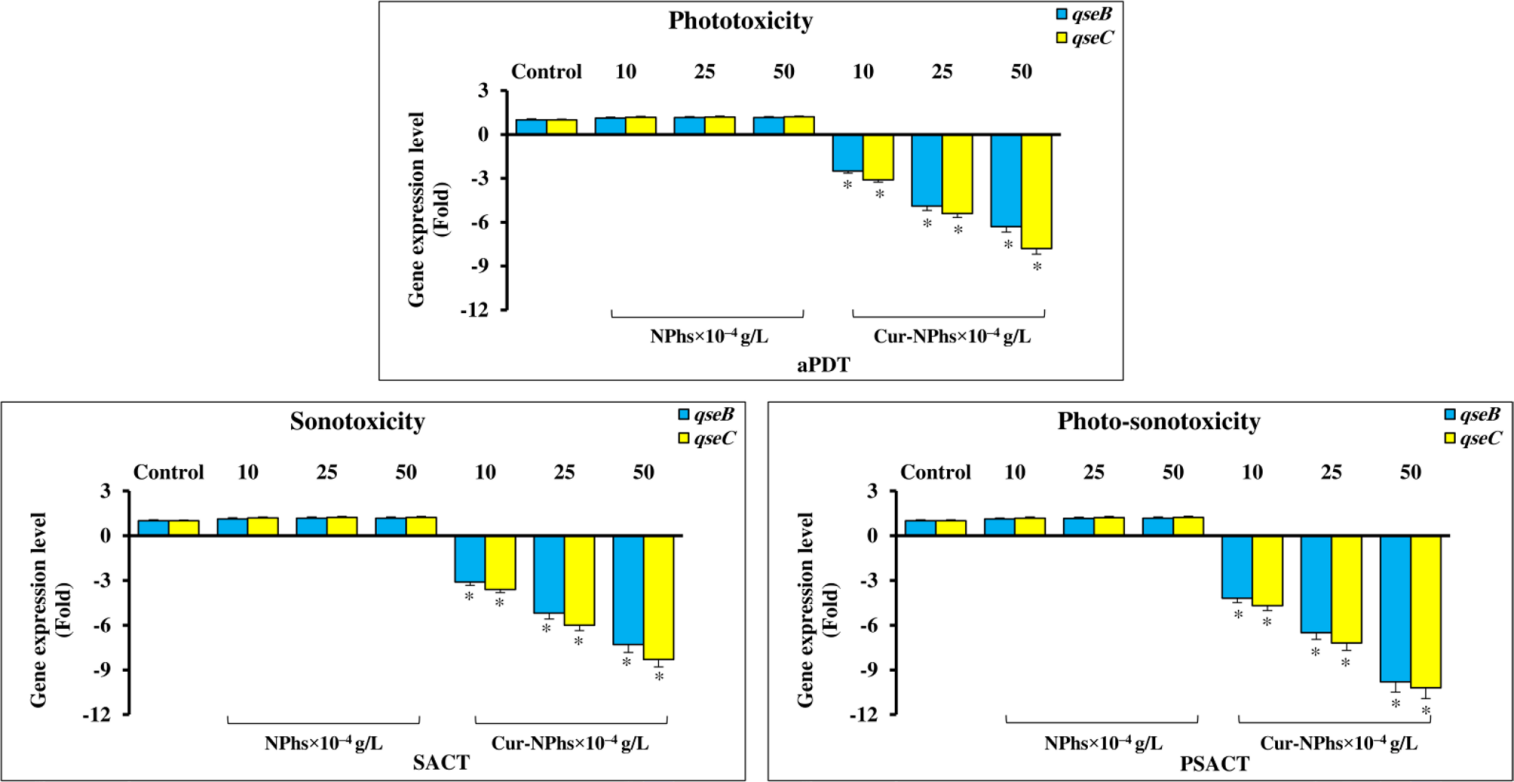
Gene expression levels of *qseB* and *qseC* following different treatments. Significant differences according to the control, *P < 0.05.

The transcript levels of the biofilm-associated genes, *qseB* and *qseC*, were measured by performing qRT-PCR experiments. This study indicated that there was a dose-dependent manner effect of the treatment on the expression levels of *qseB* and *qseC*. The results showed that the expression levels of *qseB* were downregulated 4.2-, 6.5-, and 9.8-fold in treated *A. actinomycetemcomitans* by PSACT using 10, 25, and 50 × 10^–4^ g/L Cur-NPhs, respectively (Fig. 9; P < 0.05). Corresponding values in *qseC* were 4.7-, 7.2-, and 10.2-fold, respectively (P < 0.05).

As shown in Fig. 9, there were the significant 2.5-, 4.9-, and 6.3-fold downregulation of *qseB* and 3.1-, 5.4-, and 7.8-fold downregulation of *qseC*, in response to 10, 25, and 50 × 10^–4^ g/L increased concentrations of Cur-NPhs during aPDT, respectively (P < 0.05). Also, the transcript levels of *qseB* were downregulated 3.1-, 5.2-, and 7.3-fold, respectively, in treated *A. actinomycetemcomitans* by SACT using10, 25, and 50 × 10^–4^ g/L Cur-NPhs compared with those in the transcript levels of *qseC* that were 3.6-, 6.0-, and 8.3-fold, respectively (P < 0.05). According to the data obtained in the present study, there was no significant difference in the rate of transcript levels between the two genes in treated *A. actinomycetemcomitans* with aPDT and SACT (P > 0.05). As shown in Fig. 9, there was no significant changes in gene expression of *qseB* and *qseC* following NPhs-mediated aPDT-, SACT-, and PSACT-treatment (P > 0.0.5).

### Monitoring of biofilm-associated *rcpA* gene expression

**Figure 10:**
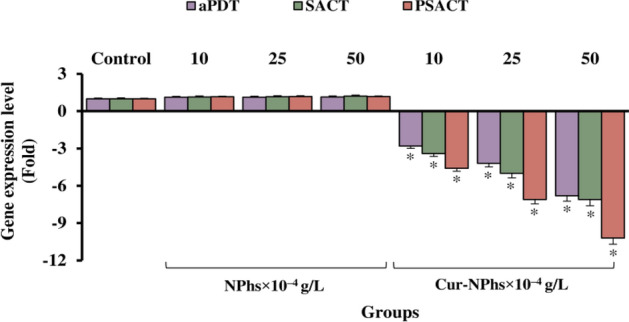
Gene expression levels of *rcpA* following different treatments. Significant differences according to the control, *P < 0.05.

Significant reductions of *rcpA* were observed at 10, 25, and 50 × 10^–4^ g/L of Cur-NPhs-aPDT-, SACT-, and PSACT-treated *A. actinomycetemcomitans* (4.4-, 5.2-, and 9.7-fold, respectively). No significant change of *rcpA* mRNA expression was observed in treated groups using NPhs-aPDT, NPhs-SACT, and NPhs-PSACT at the concentrations of 10, 25, and 50 × 10^–4^ g/L (all P > 0.05; Fig. 10).

## Discussion

PSACT can be defined as the promising approach for eradication of microbial cells through the interaction of photo-sonosensitizer as a non-toxic photo-sonosensitizing agent with visible light at specific wavelength and ultrasonic waves with specific intensity. PSACT minimizes the side effects, maximizes the responses on-target, and can be focused deeply within the target site to a single discrete point in three dimensions^11^. After the activation of photo-sonosensitizer with the appropriate wavelength of light, electrons are transferred from a low to a higher level of energy which is called the triplet state that can undergo two pathways: transfer directly to the neighboring molecule or cell membrane (type-1 reaction) to form a radical anion which reacts with oxygen to produce ROS; or transfer its energy directly to molecular oxygen (type-2 reaction) to produce excited-state singlet oxygen^12–14^. In addition, the relatively low-intensity ultrasound excites the photo-sonosensitizer at the target site, which leads to microbubble formation through the acoustic cavitation process resulting in the generation of ROS.

The characteristics of the ideal photo-sonosensitizer have been discussed previously^15^. Briefly, they should be chemical purity non-toxic, absorb the light in the red or far-red wavelengths, eliminate rapidly from normal tissue, be cost-effective and easily available, and generate a large number of cytotoxic products. Based on the literature, the photosensitizers used in the treatment of periodontitis included methylene blue (MB), indocyanine green (ICG), phenothiazine chloride, toluidine blue (TBO), and Cur^16–22^.

Curcumin longa, derived from the turmeric rhizomes (*Curcuma longa* L.), is well known for its wide range of pharmacological properties^23^. The bioavailability limitations of Cur such as its hydrophobicity, low solubility, instability, poor absorption, rapid metabolism, and fast systemic removal have led to ongoing attempts for developing a new formulation to enhance its solubility, bioavailability, and therapeutic activities^24^. Evidence from several studies has indicated that phytosomes markedly improve the bioavailability and pharmacokinetics of Cur^24,25^. As recently reported by Purpura et al.^26^, phytosomes significantly improve the relative human bioavailability of Cur by 19.2-fold for Cur alone. It has been observed that Cur is directly bound to the polar head of phosphatidylcholine in phytosomes and the covalent bonds between phosphatidylcholine and the phytochemical cargo (i.e., Cur) are the main factor in stability profile of phytosomes compared with many other delivery systems^27^. Although there are several studies that investigate the efficacy of phytosomal Cur in the treatment of cancer, diabetes, and inflammatory diseases^28–32^, no study has hitherto assessed the antimicrobial properties of Cur-decorated NPhs especially during PSACT against periopathogenic bacteria.

In the current study, Cur-NPhs alone were able to reduce the planktonic *A. actinomycetemcomitan*s counts significantly but had no inhibitory effect on biofilm growth. The observation of resistance to the Cur-NPhs when *A. actinomycetemcomitans* was grown in biofilms, suggests that biofilm formation is one of the critical virulence factors for this species, which permits *A. actinomycetemcomitans* to establish persistent periodontitis and peri-implantitis. Biofilm production by *A. actinomycetemcomitans* and its role in oral infections remain poorly studied. It has been revealed that the resistance of biofilms to antimicrobial agents is increased 10–1000 times compared with what is normally seen with planktonic bacteria. In fact, when microbial cells exist in a biofilm, may be more resistant to antimicrobial agents because the microorganisms are protected against the action of the agents, with the biofilm impairing the entry of agents by acting as a barrier for diffusion. The biofilm matrix also can react with or adsorb the antimicrobial agents, thereby reducing the number of available agents to interact with microbial cells in the biofilm structure. In addition, microbial cells in biofilm structure have reduced growth rates and metabolic activity. Another possibility that microbial cells in the biofilm are tolerant to antimicrobial agents is conjugation among biofilm cells. This exchange of genes is important for the acquisition of resistance genes and genetic diversity of bacterial communities and favors the exchange of genes that can involve in more antimicrobial resistance^33,34^.

The results of characterization of synthesized Cur-NPhs as the photo-sonosensitizer in the current study are consistent with a recent report in which strong interaction between Cur and phosphatidylcholine leads to the stability of phytosomes^26^. The significant reduction of log_10_ CFU/mL in *A. actinomycetemcomitans* (13.6 log_10_ CFU/mL reduction) following PSACT using the high concentrations of Cur-NPhs (50 × 10^–4^ g/L) combined with the blue laser irradiation at a wavelength of 450 ± 5 nm plus ultrasonic waves with the intensity of 1.56 W/cm^2^ at a frequency of 1 MHz for 2 min showed that Cur-NPhs-PSACT had an antibacterial effect. The results of this study revealed the activation of Cur-NPhs using both blue laser irradiation and ultrasonic waves enhanced the antimicrobial activity of Cur-NPhs which could be due to increased ROS and singlet oxygen. So, Cur-NPhs-PSACT had higher antibacterial activity than Cur-NPhs-aPDT and Cur-NPhs-SACT against *A. actinomycetemcomitans*. According to our results, activated NPhs by blue laser irradiation, ultrasonic waves, and both of them in NPhs-aPDT, NPs-SACT, and NPhs-PSACT treatment had no contribution to the observed antimicrobial effects. Notably, our finding that Cur-NPhs-aPDT had an antibacterial effect against *A. actinomycetemcomitans* is similar to previous reports, which show the antibacterial effect of Cur-aPDT^35^.

As previously mentioned, biofilm structures are the protected niches for *A. actinomycetemcomitans* against host defense, hostile environments conditions, and antimicrobial treatment^36,37^. Nevertheless, some antimicrobial molecules can penetrate throughout the biofilm matrix and lead to biofilm degradation. Our findings are consistent with recent studies that show aPDT using Cur as a photosensitizer is efficient in the reduction of microbial biofilms^38^. As the results of this study showed, PSACT using 50 × 10^–4^ g/L Cur-NPhs had the most effective anti-biofilm activity against *A. actinomycetemcomitans* and could degrade the biofilm cells up to 65%. In addition, the results showed that the reduction of *A. actinomycetemcomitans* metabolic activity was dose-dependent. There was a more significant reduction of metabolic activity following Cur-NPhs-mediated PSACT at the concentrations of 50 × 10^–4^ g/L about 90.0%. Additionally, aPDT and SACT with 50 × 10^–4^ g/L Cur-NPhs could decrease the metabolic activity to 70% and 78%, respectively. The finding indicated the antimetabolite activity of PSAT was more than aPDT and SACT.

The results regarding the reduction of metabolic activity of *A. actinomycetemcomitans* during aPDT treatment, corroborate with the results found in the literature for this and another pathogen^35,39,40^. Ari et al.^40^ studied the effects of bystander responses following aPDT using Cur as a photosensitizer on *A. actinomycetemcomitans* biofilm growth. Their results showed that bacterial metabolic activity significantly decreased by 42.6% after exposure to bystander effects induced by Cur-aPDT treated whole bacterial cell suspension. In the study reported by Pourhajibagher et al.^35^, the metabolic activity of treated *A. actinomycetemcomitans* by nano-chitosan encapsulated ICG-mediated aPDT was assessed. Based on their results, a statistically significant reduction (48%) was observed in the metabolic activity of *A. actinomycetemcomitans*.

The QseBC two-component systems are characterized as the global regulators of virulence. QseBC is associated with QS and essential for *A. actinomycetemcomitans* to adapt and respond to various environmental stimuli^41^. As Novak et al.^42^ reported, QseBC contributes to the regulation of colonization, biofilm formation, and expression of virulence genes in *A. actinomycetemcomitans*. Based on the QseBC signaling cascade of *A. actinomycetemcomitans*, the expression of *qseBC* in *A. actinomycetemcomitans* is induced by exogenous autoinducer-2 (AI-2), which may occur via the motility QS regulator^43^. Also, the expression of *rcpA* has been linked to biofilm production. RcpA protein has been demonstrated to be involved in outer membrane channel formation that allows for the secretion of the assembled fimbriae to the microbial cell surface. Fimbriae in this species are responsible for biofilm formation which ultimately lead to increased antimicrobial resistance^44^.

Weigel et al.^43^ reported the functional outcomes of *A. actinomycetemcomitans* QseBC have been defined as influencing biofilm growth and virulence. Also, the findings of Novak et al.^42^ study indicated that *qseBC* is require for stimulation of biofilm formation of *A. actinomycetemcomitans* in an animal model of periodontitis. In this study, both *qseB* and *qseC* genes were significantly downregulated after treatment with Cur-NPhs-aPDT-, SACT-, and PSACT-treated *A. actinomycetemcomitans* Cur-NPhs-mediated PSACT compared to the other control groups. Interestingly, the downregulation of *qseB* and *qseC* on treated *A. actinomycetemcomitans* were dose-dependent and may be involved in the attenuation of *A. actinomycetemcomitans* QS.

Moreover, in the current study, after exposing the biofilm of *A. actinomycetemcomitans* strains to Cur-NPhs-PSACT, *rcpA* gene expression was potentially downregulated in a dose-dependent manner which may be the result of the accumulation of Cur-NPhs in the biofilm environment. Since the *rcpA* gene plays an important role in biofilm formation in *A. actinomycetemcomitans*, the expression inhibition of this gene following Cur-NPhs-PSACT treatment may cause to decrease bacterial adhesion, biofilm formation, and its pathogenicity. Various recent reports have shown that aPDT can overcome bacterial biofilm formation through affecting *rcpA* gene expression in *A. actinomycetemcomitans*^6,35^. Pourhajibagher et al.^35^ investigated the efficacy of Cur-aPDT as an alternative antimicrobial approach against *A. actinomycetemcomitans*. Their results indicated aPDT with Cur leads to significantly decreased *A. actinomycetemcomitans* cell survival and reduced the expression of the *rcpA* gene by approximately 8.5-fold. In another study, the outcome of the expression level of *A. actinomycetemcomitans rcpA* gene following treatment by ICG-aPDT was investigated. The results showed that ICG-mediated aPDT using 125 μg/mL ICG at the fluency of 15.6 J/cm^2^ of diode laser irradiation could significantly reduce the expression of *A. actinomycetemcomitans rcpA* gene approximately sixfold^6^. According to the results of the current study, no change in *rcpA*, *qseB*, and *qseC* mRNA expressions in either of the NPhs and NPhs-treated groups during aPDT-, SACT-, and PSACT-treatment suggests these treatments have no effect on expressions of test genes and therefore on biofilm production in *A. actinomycetemcomitans*.

## Conclusion

This study concludes that the Cur-NPhs-PSACT could reduce the cell viability, metabolic activity, and biofilm growth in *A. actinomycetemcomitans* by downregulating the expression of *rcpA*, *qseB*, and *qseC* genes, and may attenuate pathogens that decrease disease severity in patients with periodontitis and peri-implantitis. Our findings warrant detailed examination of the interactions between the Cur-NPhs-PSACT as an adjunctive therapy and pathogenicity of the main periodontal pathogens toward efforts to successful treatment of periodontal infections.

## Materials and methods

### Materials

Cur and phosphatidylcholine were purchased from Sigma-Aldrich, Steinheim, Germany. Dichloromethane was obtained from Merck, Darmstadt, Germany. Brain heart infusion (BHI) agar and BHI broth were purchased from Conda, Torrejon de Ardoz, Spain. Yeast extract, hemin, and menadione were obtained from Merck, Darmstadt, Germany. Phosphate buffered saline (PBS), crystal violet (CV), and acetic acid were purchased from Sigma-Aldrich, Steinheim, Germany. GeneAll Hybrid-R RNA purification kit was purchased from GeneAll Biotechnology Co., Korea. RNase-free DNase I and Revert Aid First Strand cDNA Synthesis Kits were purchased from Thermo Fisher Scientific, US. SYBR Green master mix was obtained from Takara, Kyoto, Japan. Cell Proliferation Kit II (XTT) was obtained from Sigma-Aldrich, Steinheim, Germany. Ethanol and other reagents were all of the analytical grade.

### Preparation of Cur-decorated NPhs

The NPhs preparation was done by de-hydration and re-hydration technique of Abdul Azeez et al.^45^ with minor modifications. Briefly, 0.2 g of phosphatidylcholine was dissolved in 20 mL of dichloromethane as an organic solvent and stored for 2 h. The dichloromethane was then removed under a reduced pressure and temperature using a rotary vacuum evaporator. A thin layer containing phytosome would be formed at the bottom of the flask. After that, the thin layer of phytosome is re-hydrated with distilled water to form micelles that are then probe sonicated to achieve NPhs.

Cur-NPhs were prepared according to the method reported recently with slight modification^46^. In brief, Cur and prepared NPhs in the molar ratio of 1:1 was dissolved in 20 mL of dichloromethane. The mixtures were thoroughly mixed at room temperature on a magnetic stirrer and the solvent was evaporated under reduced pressure in a rotary evaporator. The obtained thin film was then washed with *n*-hexane, dried under vacuum, hydrated for 2 h, and ultrasonicated to obtain Cur-NPhs. The resultant Cur-NPhs were placed in amber-colored glass bottle and stored at room temperature.

### Characterization of Cur-NPhs

#### Morphology

The surface morphology, size, and shape of prepared Cur-NPhs were confirmed using a field emission scanning electron microscope (FESEM; Zeiss, Sigma VP, Germany, 15 kV accelerating voltage). Moreover, the core–shell structure of Cur-NPhs was assessed by transmission electron microscope (TEM; Zeiss EM10C) with an accelerating voltage of 100 kV.

#### Measurement of particle size, polydispersity, and ζ-potential

The Cur-NPhs dispersion was diluted with PBS solution prior analysis. Average particle size, polydispersity, and ζ-potential of Cur-NPhs were determined using a dynamic light scattering particle size analyzer (Malvern Zetasizer Nano ZS system) at 25 °C with a scattering angle of 90°.

#### Entrapment efficiency (EE)

The percentage of Cur incorporated was determined by centrifuging the Cur-NPhs at 8000 rpm for 30 min. The supernatant was then obtained to measure the absorbance at 425 nm using a UV–visible spectrophotometer. The encapsulation efficiency (EE) was determined using the following equation:

$${\text{EE}}\%=\frac{{\text{Initial Cur }} ({\text{g}})-{\text{ Free Cur }}({\text{g}})}{{\text{Initial Cur }}({\text{g}})} \times 100$$

#### In vitro drug release

The release properties of Cur from Cur-NPhs were obtained as described earlier^47^. The absorbance of Cur was measured using the UV–visible spectrophotometer at 426 nm. The percentage of released Cur was calculated as:

$${\text{Drug release}}\%=\frac{{\text{Cur released }}({\text{mg}}/{\text{mL}})}{{\text{Cur decorated }}({\text{mg}}/{\text{mL}})} \times 100$$

#### Physical stability of Cur-NPhs during storage

To evaluate shelf life, Cur-NPhs was stored in the containers at 4 °C and 37 °C in the dark. Changes in the polydispersity index, particle size, and ζ-potential were investigated at different time intervals during storage for 2 weeks. Effects of different pH (pH = 5, 6, 7, 8, 9) on the retention rate (RR) of Cur-NPhs was determined after 24 and 48 h of storage. RR of Cur was measured at 425 nm and calculated as follows:

$${\text{RR}}\%=\frac{{\text{Cur at }}{t}_{0}}{{\text{Cur at }}{t}_{1}} \times 100$$

### Bacterial strain and growth conditions

*A. actinomycetemcomitans* IR-TUMS/BPG4 (Accession number in Genbank: KX108928) was grown at 37 °C in a capnophilic condition (3–5% CO_2_ and 8–10% O_2_) in BHI broth supplemented with 0.6% yeast extract and 1 mg/L menadione^48^.

### Light source

A continuous blue laser (ASHA, Iran) at the wavelength of 450 ± 5 nm with an output intensity of 150 mW/cm^2^, 4.2 V, and 0.34 A was used as a light source. The blue laser was placed approximately 1 mm away from the surface of the bacterial suspension.

### Ultrasound system

SACT testing was done as described previously with slight modification^49^. In brief, a flat microtiterplate containing the bacterial suspension was held in a plastic holder tight to the bottom of a closed dark chamber of an ultrasonic bath containing cooled distilled water. This was done to prevent any external illumination. Sonication was conducted at a frequency of 1 MHz and pulsed repetition frequency of 100 Hz with a spatial average ultrasonic intensity of 2 W/cm^2^ for 5 min. An ultrasound transducer with a surface area of 7.0 cm^2^ was applied at the bottom of the chamber of the ultrasonic bath to provide exposure to the bacterial suspension.

### Experimental design

To determine the antimicrobial response to Cur-NPhs-mediated PSACT, test groups consisted of *A. actinomycetemcomitans* subjected to:

1. NPhs: at the concentrations of 10, 25, and 50 × 10^–4^ g/L.
2. Cur-NPhs: at the concentrations of 10, 25, and 50 × 10^–4^ g/L.
3. Blue laser: irradiation at a wavelength of 450 ± 5 nm for 2 min.
4. Ultrasonic waves: the intensity of 2 W/cm^2^ for 2 min at a frequency of 1 MHz.
5. NPhs-aPDT: NPhs (10, 25, and 50 × 10^–4^ g/L) + blue laser irradiation at a wavelength of 450 ± 5 nm for 2 min.
6. Cur-NPhs-aPDT: Cur-NPhs (10, 25, and 50 × 10^–4^ g/L) + blue laser irradiation at a wavelength of 450 ± 5 nm for 2 min.
7. NPhs-SACT: NPhs (10, 25, and 50 × 10^–4^ g/L) + ultrasonic waves with the intensity of 1.56 W/cm^2^ for 2 min at a frequency of 1 MHz.
8. Cur-NPhs-SACT: Cur-NPhs (10, 25, and 50 × 10^–4^ g/L) + ultrasonic waves with intensity of 1.56 W/cm^2^ for 2 min at a frequency of 1 MHz.
9. NPhs-PSACT: NPhs (10, 25, and 50 × 10^–4^ g/L) + blue laser irradiation at a wavelength of 450 ± 5 nm + ultrasonic waves with the intensity of 1.56 W/cm^2^ at a frequency of 1 MHz for 2 min.
10. Cur-NPhs-PSACT: Cur-NPhs (10, 25, and 50 × 10^–4^ g/L) + blue laser irradiation at a wavelength of 450 ± 5 nm + ultrasonic waves with the intensity of 1.56 W/cm^2^ at a frequency of 1 MHz for 2 min.
11. Control group (bacterial suspension without treatment).

### aPDT protocol

To find out the effect of aPDT based on NPhs and Cur-NPhs at different concentrations (10, 25, and 50 × 10^–4^ g/L; final concentration), 100 μL of each concentration of test materials was added to the well of a 96-well microtiterplate. The wells were then inoculated with a 100 μL/well of fresh BHI broth bacterial cultures (adjusted to the final concentration of 5.0 × 10^5^ CFU/mL). The microtiterplate was incubated for 5 min in the dark at 25 °C. The NPhs and Cur-NPhs treated bacterial suspensions in microtiterplate wells were immediately exposed with the blue laser irradiation at a wavelength of 450 ± 5 nm for 2 min.

### SACT protocol

SACT was performed as described previously^50^. Briefly, 100 μL of NPhs and Cur-NPhs at different concentrations (10, 25, and 50 × 10^–4^ g/L; final concentration) separately was added to the well of a 96-well microtiterplate. The wells were then inoculated with a fresh BHI broth bacterial suspension (100 μL/well; final concentration of 5.0 × 10^5^ CFU/mL). The microtiterplate was incubated in the dark room (5 min; 25 °C). After that sonication was done with an ultrasound transducer containing a surface area of 7.0 cm^2^ at a pulsed repetition frequency of 100 Hz.

### PSACT protocol

**Figure 11:**
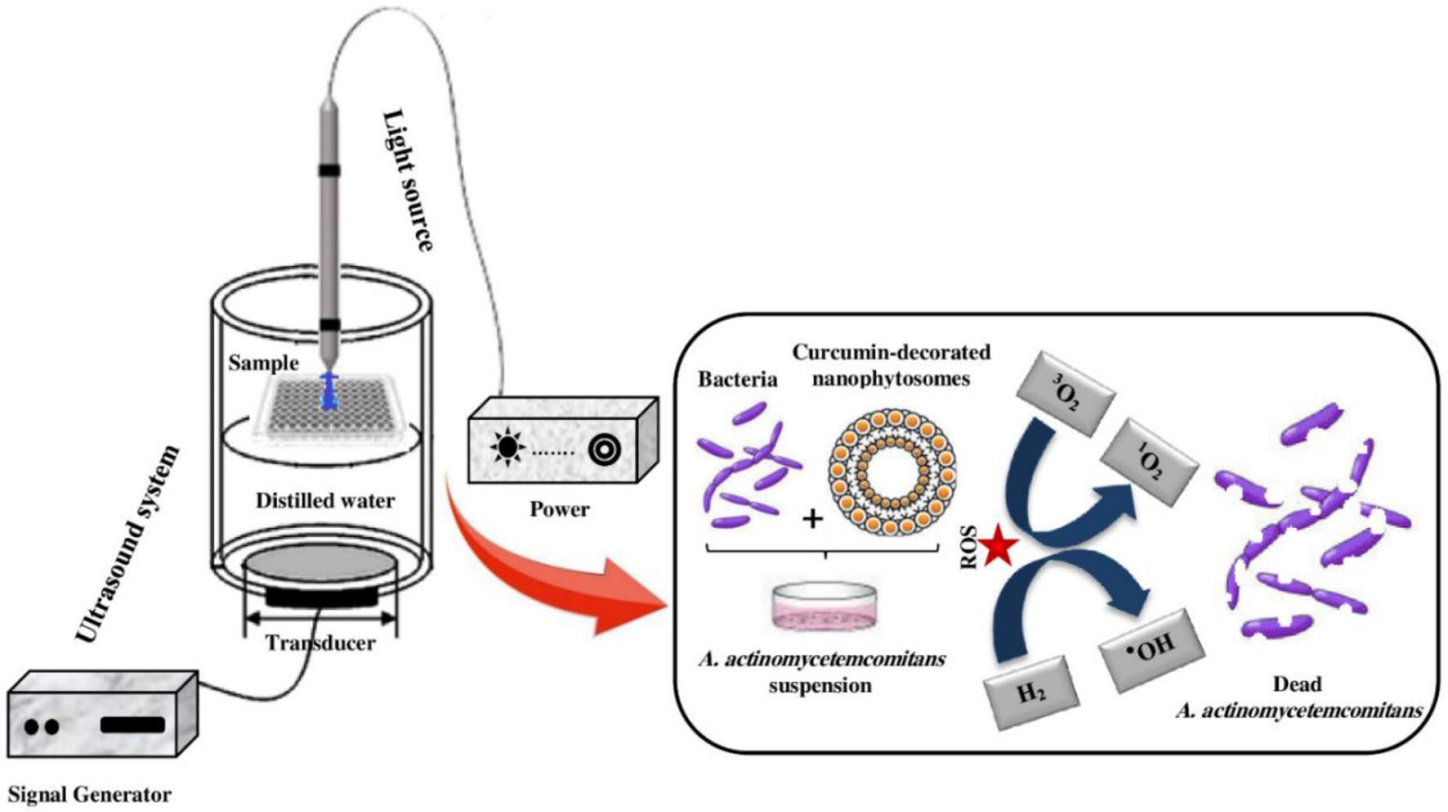
Schematic apparatus for illumination treatment of sample.

100 μL of bacterial suspension (1.5 × 10^6^ CFU/mL) was transferred to the wells of the 96-well microtiterplate. 100 μL of NPhs and Cur-NPhs at different concentrations (10, 25, and 50 × 10^–4^ g/L) were separately added to the bacterial suspension. After incubation of the microtiter plate in a dark capnophilic atmosphere for 5 min at room temperature, the bacterial suspension was simultaneously exposed to blue laser at the wavelength of 450 ± 5 nm with an output intensity of 150 mW/cm^2^ and ultrasonic waves at a frequency of 1 MHz with an ultrasonic intensity of 2 W/cm^2^ for two min. The schematic diagram of PSACT is shown in Fig. 11.

### Determination of viability of *A. actinomycetemcomitans* in planktonic culture following treatment groups

100 μL of NPhs and Cur-NPhs (both at the concentrations of 200 × 10^–4^ g/L) were added to the well in column one of a 96-well microtiterplate, separately, and was diluted two-fold stepwise to column three after adding 100 μL of 2 × BHI broth to each well. Then, an *A. actinomycetemcomitans* suspension (100 μL of 1.5 × 10^6^ CFU/mL) was poured into each well. In this experiment, the concentration of test materials was in the range of 10, 25, and 50 × 10^–4^ g/L. Then microtiterplate was incubated for 24 h under capnophilic condition, in the dark, at 37 °C. Thereafter, 50 μL of each well were cultured in a BHI agar plate supplemented with 0.6% (wt/vol) yeast extract, 5–7% defibrinated sheep blood, 5 mg/L hemin, and 1 mg/L menadione. The BHI agar plates were incubated in a capnophilic condition at 37 °C for 48 h, and the number of CFU/mL was determined.

The effect of NPhs- and Cur-NPhs-mediated aPDT-, SACT-, and PSACT-treatment on *A. actinomycetemcomitans* in planktonic culture were done according to the method mentioned as previously^42^. Briefly, NPhs and Cur-NPhs (both 100 μL; 200 × 10^–4^ g/L) were added to the well in column one of a 96-well microtiterplate, separately, and was diluted two-fold stepwise to column three after adding of 2 × BHI broth (100 μL) to each well. Then, an *A. actinomycetemcomitans* suspension (100 μL of 1.5 × 10^6^ CFU/mL) was added into each well. The microtiterplate was incubated for 5 min in the dark, at 25 °C. The contents of microtiterplate wells were immediately treated in aPDT-, SACT-, and PSACT-protocols as mentioned in the experimental design section. As mentioned in the above paragraph, the number of CFU/mL of treated *A. actinomycetemcomitans* in planktonic culture was determined.

### Determination of treatment groups efficiency on biofilm killing/degradation

The biofilm killing/ degradation effect of NPhs- and Cur-NPhs-mediated aPDT-, SACT-, and PSACT-treatment was determined according to a previous study^51^. Briefly, aliquots in BHI broth medium of bacterial suspensions (200 µL; 1.0 × 10^5^ CFU/mL final concentration) were transferred into each well in 96-well microtiterplate. To maturation biofilm of *A. actinomycetemcomitans* then microtiterplate was incubated for 48 h in capnophilic condition, at 37 °C. After that, the medium was removed from each well, and planktonic cells were deducted by washing three times with PBS (pH 7.4). *A. actinomycetemcomitans* cells in biofilms were treated in aPDT-, SACT-, and PSACT-protocols as mentioned in the experimental design section.

After each treatment, biofilm killing/degradation was assessed with the colorimetric method based on CV assay^52^. The content of each well of microplates was removed and the planktonic cells were deducted were allowed to air dry at room temperature. Then, the wells were stained with CV dye (1%) for 20 min at 25 °C, and the dye was discarded and washed twice with PBS (pH 7.4). Ethanol (200 μL; 95%) was added into each well and was incubated for 15 min at 25 °C. Then, the content of each well quite was aspirated and the microtiterplate was allowed to air dry at room temperature. Thereafter, each well was filled with acetic acid (200 μL; 33%) and biofilms were quantified by measuring the optical densities (OD) value using a microplate reader machine (at 570 nm; Thermo Fisher Scientific, US). To assess the treatment efficiency of NPhs- and Cur-NPhs-mediated aPDT-, SACT-, and PSACT-efficiency on biofilm killing/degradation, the percentage of biofilm killing/degradation was determined by the following equation:

$${\text{Biofilm killing}}/{\text{degradation}}\%=\frac{{\text{OD of untreated }}{\it A}.\, {\it actinomycetemcomitans}-{\text{ OD of sample}}}{{\text{OD of untreated }}{\it A.}\, {\it actinomycetemcomitans}} \times 100$$

### Assessment of metabolic activity of treatment groups using XTT reduction assay

The metabolic activity of treated *A. actinomycetemcomitans* was assessed using the XTT (2,3-bis [2-methyloxy-4-nitro-5-sulfophenyl]-2H-tetrazolium-5-carboxanilide) reduction assay, as described by Coraça-Hubér et al.^53^. Following each treatment described in the experimental design section, the content of wells was collected and centrifuged at 2000 rpm for 10 min. The supernatants were removed and resulting bacterial cell sediments were dissolved in 150 μL of XTT-menadione-PBS solution in microplate wells and incubated at 37 °C. After 3 h, 100 μL of the mixture was transferred to a new microplate and the optical intensity was measured at 492 nm using a microplate reader.

### Determination of relative quantification of quorum-sensing-associated *qseB* and *qseC* genes expression

To determine the effects of treatment groups on QS genes expression in *A. actinomycetemcomitans*, the treated *A. actinomycetemcomitans* was used to extract RNA using GeneAll Hybrid-R RNA purification kit following the manufacturer’s instructions. The extracted RNA was quantified spectrophotometrically using a NanoDrop spectrophotometer Thermo Fisher Scientific, US) and the quality was confirmed on the agarose gel. Total RNA was pretreated with RNase-free DNase I treatment according to the manufacturer's protocol to remove any residual chromosomal DNA. The first-strand cDNA was synthesized from the total RNA using a Revert Aid First Strand cDNA Synthesis Kit according to the manufacturer’s protocol. Quantitative real-time polymerase chain reaction (qRT-PCR) was then performed to quantify gene transcription of *qseB* and *qse*C. The nucleotide sequences of primers used in this study were shown in Table 1. The specificity of the primers was evaluated on agarose gel electrophoresis. Reaction plates were processed on the Line-GeneK Real-Time PCR Detection System and Software (Bioer Technology, Hangzhou, China) using SYBR Green master mixes with the following cycling parameters: initial denaturation at 95 °C/2 min, followed by 40 cycles at 94 °C/10 s, 60 °C/10 s, and 72 °C/10 s. Finally, the expression level of *qseB* and *qse*C genes was analyzed based on the previous study^54^. *16S rRNA* was used as an internal control to normalize RNA concentration.

**Table 1:** Primer sequences used in this study.

Gene	Primer	Sequence (5′→3′)	Length	Size (bp)
*qseB*	Forward	GCAGTGGTGCTGGATTTAACCTTG	24	157
	Reverse	GCGTTACTGCTCACTTCGTTATCCC	25	
*qseC*	Forward	TAAGTGGAATAATTACAGCCTGCG	24	176
	Reverse	TTGTTGTGCGTCAAACACTTGGTTC	25	
*rcpA*	Forward	GGGCATTAACTGGAGCCAC	19	72
	Reverse	ATCCACCTCCGAAACCGAAG	20	
*16S rRNA*	Forward	GTGAAATCCCCGGGCTTAAC	20	217
	Reverse	ACCGTTTACAGCGTGGACTA	20	

### Determination of relative quantification of biofilm-associated *rcpA* gene expression

The evaluation of *rcpA* expression was performed based on the previous study^42^. Total RNA extraction, cDNA synthesis, and qRT-PCR was done as described above (Section Determination of relative quantification of quorum-sensing-associated *qseB* and *qseC* genes expression).

### Statistical analyses

All experiments were performed in triplicate and expressed as mean ± standard deviation (SD). All quantitative data were analyzed with one-way analysis of variance (ANOVA) and Tukey test using the SPSS software version 23.0. The significance level was set at P < 0.05. Fold differences in RNA expression were determined by the 2^−ΔΔCt^ method and the changes greater than or equal to two-fold were considered significant^54^.
